# ECM-Like Scaffolds: Nature Drives Research

**DOI:** 10.1155/2014/298361

**Published:** 2014-12-28

**Authors:** Costantino Del Gaudio, Silvia Baiguera, Alessandra Bianco, Luca Urbani

**Affiliations:** ^1^Department of Enterprise Engineering, Intrauniversitary Consortium for Material Science and Technology (INSTM), Research Unit “Tor Vergata”, University of Rome “Tor Vergata”, Via del Politecnico 1, 00133 Rome, Italy; ^2^INAIL-DIPIA, Via Alessandria 220E, 00198 Rome, Italy; ^3^Surgery Unit, Institute of Child Health and Great Ormond Street Hospital, University College London, 30 Guilford Street, London WC1N 1EH, UK

The development of an* ad hoc* scaffold for effective tissue engineering applications plays a pivotal role in promoting and support the healing process. For this aim, an accurate morphological, biochemical, and mechanical replication of the tissue-specific extracellular matrix (ECM) to be regenerated can contribute to elicit a positive cell response, tissue formation and, therefore, a proper host integration after implantation. To further enhance this expected result, several technical strategies can be usefully planned, including innovative fabrication techniques, surface modification, incorporation of specific fillers, and/or addition of drugs or growth factors to be subsequently released to speed up the autologous tissue regeneration.

This special issue presents both original research and review articles aimed to investigate and underline the crucial role of an ECM-like scaffold. In order to present an overview on the* in vivo* results collected so far, according to tissue engineering guidelines, S. Baiguera et al. focused on the analysis of implanted orthotopic organ substitutes. Different clinical fields were considered, highlighting limitations and promising approaches when properly prepared scaffolds were used. M. G. de Morais et al. reviewed the potential role of* Spirulina*, a prokaryotic microalga, as a specific element to be included into electrospun polymeric fibers to mimic the architecture of natural ECM. A key topic for an effective tissue regeneration was considered by A. Neve et al., emphasizing the role played by ECM in the regulation of the angiogenic process. This is a well-known issue to be addressed in order to deal with a suitable tissue engineered construct, and the proposed review also shows that ECM molecules and fragments, resulting from proteolysis, can act directly as inflammatory stimuli and contribute to exacerbated angiogenesis.

Original research studies demonstrate the influence of natural cues for the fabrication of tailored constructs. E. Stocco et al. propose a hybrid scaffold made of polyvinyl alcohol hydrogel and ECM to promote cartilage regeneration. Similarly, H. Fan et al. combined human-like collagen and nanohydroxyapatite to fabricate a specific scaffold for bone tissue engineering. Both the approaches show that a scaffold not only is a “simple” (passive) structural support but plays an active part in the healing process, supporting cell adhesion, migration, and proliferation by means of different signalling cues. More directly, L. Dall'Olmo et al. address the relevant issue to fabricate functional small-diameter vascular grafts, evaluating the potential of decellularized rat iliac arteries.* In vivo* investigation led to unsatisfactory results since the lack of endothelial cells contributed to thrombus formation and intimal proliferation. Once again, this study supports the need (i) to deeply analyse the properties of the biomaterial to be used for tissue engineering scaffolding and (ii) to understand that several factors concur to a positive outcome.

Interestingly, two papers focus on the analysis of the morphological/structural properties of human trabecular bone tissue (F. Marinozzi et al.) and composite scaffolds made of chitosan/gelatin blend and bioactive glasses for bone tissue engineering (D. Massai et al.). The proposed studies can contribute to better investigate local features of ECM and, therefore, to design biomimetic porous ECM-like scaffolds that resemble the tissue of interest.

Tissue engineering can positively improve clinical treatments, but the expected outcome is strictly dependent on the strategy to be adopted. The present special issue aims to underline this crucial aspect and, thanks to the authors participating in this project, we hope to offer a sound basis to enhance regenerative protocols.

*Costantino Del Gaudio* *Silvia Baiguera* *Alessandra Bianco* *Luca Urbani*

